# Evaluating the Phytohormone Proficiencies of Multifarious *Bacillus rugosus* for Growth Promotion in *Arachis hypogaea* (L.)

**DOI:** 10.1002/jobm.70011

**Published:** 2025-04-03

**Authors:** Aniruddh Rabari, Janki Ruparelia, Chaitanya Kumar Jha, Kahkashan Perveen, Abhijit Debnath, Maheswari Behera, Andrea Mastinu

**Affiliations:** ^1^ Department of Microbiology and Biotechnology Gujarat University Ahmedabad India; ^2^ Microbiology Department Gujarat Arts and Science College, Ellisbridge Ahmedabad India; ^3^ Department of Botany & Microbiology College of Science King Saud University Riyadh Saudi Arabia; ^4^ Krishi Vigyan Kendra Dhalai India; ^5^ Department of Botany College of Basic Science and Humanities Odisha University of Agriculture and Technology Bhubaneswar India; ^6^ Department of Molecular and Translational Medicine, Division of Pharmacology University of Brescia Brescia Italy

**Keywords:** cytokinin, gibberellin, groundnut, HCN, IAA, mineral solubilization, plant growth promotion

## Abstract

The application of Plant Growth‐Promoting Rhizobacteria (PGPR) in agriculture is increasingly emphasized as a sustainable alternative to hazardous agrochemicals. This study aimed to isolate and characterize PGPR strains from the rhizospheric soil of *Arachis hypogaea* L., hypothesizing that the rhizosphere of a healthy plant harbors beneficial microbes with significant plant growth‐promoting (PGP) attributes. The isolate AB1 demonstrated promising PGP traits, including phosphate solubilization (56.44 µg mL⁻¹), zinc solubilization (6.1 µg mL⁻¹), ammonia production (3.8 µM µg mL⁻¹), and the synthesis of hydrogen cyanide (HCN) and phytohormones. Objectives included identifying these traits and evaluating their impact on the growth of *Arachis hypogaea* L. Phytohormonal profiling of AB1 through Gas Chromatography‐Mass Spectrometry (GC‐MS) and Fourier Transform Infrared Spectroscopy (FTIR) confirmed indole fractions with characteristic peaks at 3338 cm⁻¹ (N–H stretching), 1641 cm⁻¹ (C–N bond of the indole ring), and 2984 cm⁻¹ (C–H aromatic stretching). Cytokinins and gibberellins were also detected. Molecular, physiological, and biochemical analyses identified the isolate as *Bacillus rugosus* AB1, with gene sequences deposited under GenBank accession number MZ373174. The present study is the first report of *Bacillus rugosus* AB1 as a PGPR, showcasing multifaceted PGP traits that significantly enhanced root and shoot growth, biomass, and chlorophyll content in *Arachis hypogaea* L., demonstrating its potential as a biofertilizer for sustainable agriculture.

**Abbreviations**
FTIR: Fourier transform infrared spectroscopy
GAs: Gibberellins
GC‐MS: Gas Chromatograph Mass Spectrophotometry
HCN: Hydrogen cyanide
IAA: Indole‐3‐acetic acid
NA: Nutrient Agar
NIST: National Institute of Standards and Technology
P: Phosphate
PCA: Principle component Analysis
PGP: Plant growth promoting
PGPR: Plant Growth Promoting Rhizobacteria
PVK: Pikovskaya

## 1 Introduction

Phytohormones are organic chemicals produced in minimal quantities within specific regions of plants and then transmitted to other parts, where they influence various physiological processes. Despite their small size and structural differences, phytohormones mediate plant growth and development [1]. Bacteria that enhance plant growth by producing phytohormones and other beneficial traits, such as mineral solubilization, are plant growth‐promoting rhizobacteria (PGPR) [2, 3]. Besides directly promoting plant growth, PGPR exhibits various effects that contribute to increased crop yields. These effects include enzyme production (e.g., catalase, glucanase, cellulase, chitinase), metabolite production (e.g., HCN, siderophore, ammonia), and other biocontrol traits [4, 5, 6, 7, 8, 9].

Conversely, the excessive and imbalanced use of chemical fertilizers can harm plants and beneficial microbes, leading to decreased soil fertility. Moreover, the excessive use of chemical fertilizers may result in plant toxicity and indirectly impact human health (Hamid et al. 2021). Therefore, it is crucial to balance fertilizer application to ensure optimal plant growth while preserving soil health and minimizing adverse environmental and human health effects.

Compared to the adverse effects of chemical fertilizers, using plant growth‐promoting bacteria (PGPB) as biofertilizers offers a promising solution for restoring the natural fertility of soil. Biofertilizers enrich organic matter, enhancing soil structure, texture, and fertility [10]. Additionally, they play a vital role in expanding the healthful properties of vegetables and cereals. This is achieved by enhancing antioxidant activity and increasing the content of total phenolic compounds, thereby improving the nutritional quality of crops [11, 12].

Auxins serve as primary metabolites in various plant tropisms such as phototropism, hydrotropism, and geotropism. They also play a pivotal role in promoting cell elongation while inhibiting the growth of lateral buds, thereby sustaining apical dominance [13, 14]. Gibberellins (GAs), diterpenoid acids synthesized by specialized plant growth‐promoting rhizobacteria (PGPR) strains, are primarily concentrated in actively growing plant organs. This group of phytohormones regulates many developmental processes, including stem elongation, germination, dormancy, flowering, flower development, and leaf and fruit senescence [15, 16]. Cytokinins represent the third group of phytohormones found in plants, which promote cytokinesis (cell division) in both plant roots and shoots. Additionally, they are involved in the growth and differentiation of cells. Applying cytokinins in agricultural fields has enhanced crop yield [17].

Groundnuts, or peanuts (*Arachis hypogaea* L.), are a critical oilseed crop in rain‐fed regions of Asia and Africa [2]. However, soil fertility poses a significant limitation for achieving large shell yields in these areas, leading most farmers to resort to chemical fertilizers, which often exacerbate soil degradation [18, 19]. In contrast, organic farming methods employing plant growth‐promoting rhizobacteria (PGPR) offer a promising avenue for soil rejuvenation [10, 20, 21]. The present study primarily focuses on exploring the phytohormone proficiencies of *Bacillus rugosus* AB1 in conjunction with its plant growth‐promoting (PGP) attributes. By elucidating the capabilities of *Bacillus rugosus* AB1, the research aims to contribute to sustainable agricultural practices, particularly in regions where soil fertility presents a significant challenge to crop productivity.

## 2 Materials and Methods

### 2.1 Collection of Rhizospheric Soil

**Figure 1:**
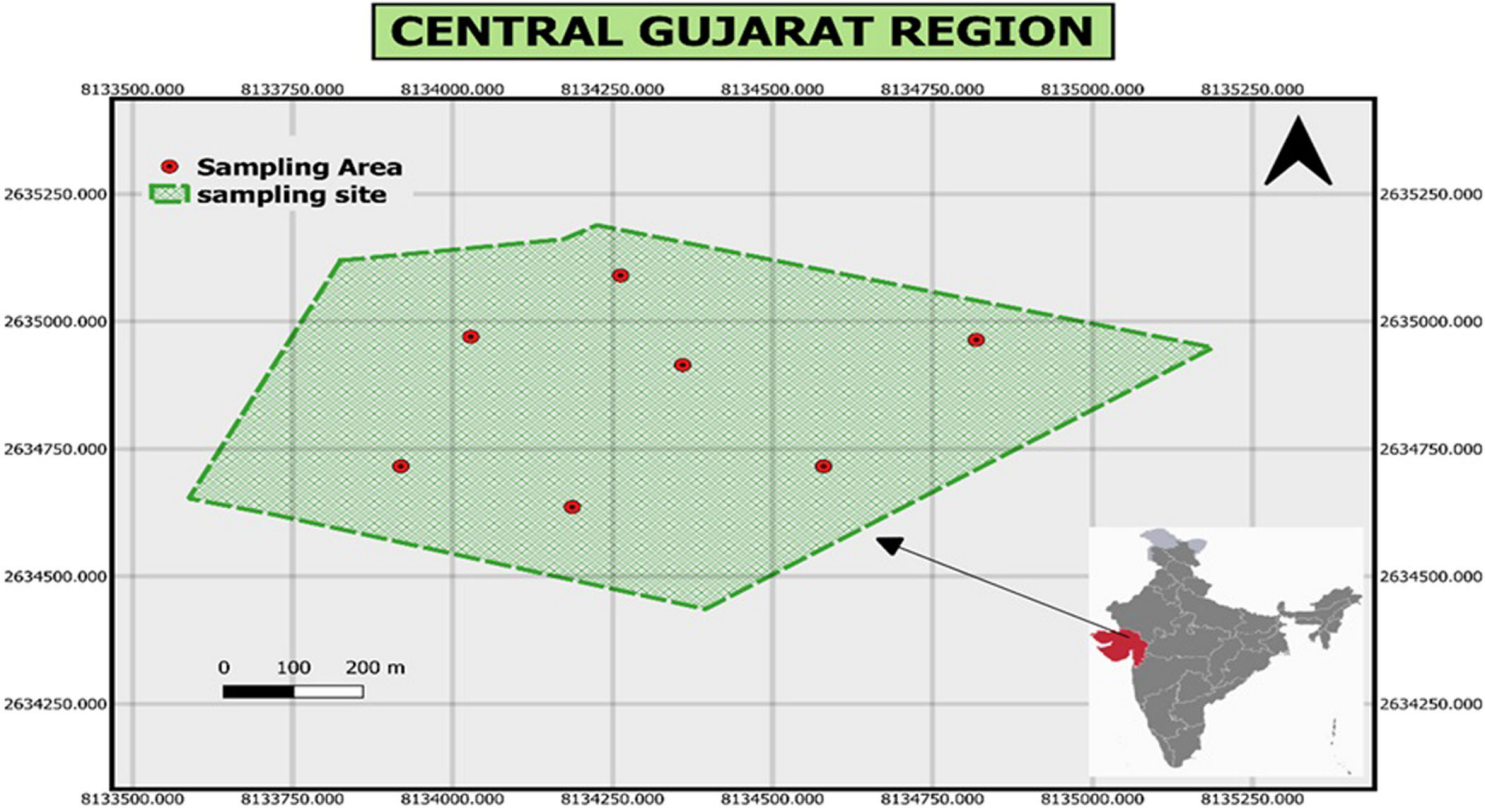
Location of sampling site.

Soil samples were collected from the rhizosphere of groundnut plants in the central Gujarat region of India, as depicted in (Figure 1). The samples were obtained from agricultural land at 6–12 cm depth. The precise geographic coordinates of the sample collection site are latitude 22.994232003470724 and longitude 73.08000665405885. Upon collection, the soil samples were carefully placed in sterile bags to maintain aseptic conditions. Subsequently, they were stored at 4°C until further analysis and experimentation. This careful handling and storage procedure ensured the preservation of microbial populations and soil characteristics for subsequent research activities [21].

### 2.2 Isolation of Plant Growth‐Promoting Bacteria

The isolation of plant growth‐promoting bacteria (PGPB) commenced with the suspension of 1 g of rhizospheric soil in 10 mL of sterile distilled water. Serial dilutions were then performed up to 10^‐5^. From each dilution, 0.1 mL of the supernatant was spread onto Nutrient Agar (NA) plates. Subsequently, the NA plates were inverted and incubated at 37°C for 24 h [22].

### 2.3 Plant Growth‐Promoting Traits of Selected Isolates

#### 2.3.1 Phosphate (P) Solubilization

A qualitative assay for phosphate (P) solubilization was conducted using tricalcium phosphate in Pikovskaya's agar medium (PVK) (having tricalcium phosphate as substrate). To screen potential isolates, spot inoculation of the desired isolates was performed in the four subdivisions of the PVK medium. The final pH of the medium was adjusted to 7. Following inoculation, the plates were incubated at 28°C for 24–48 h. Phosphate solubilization was tested in the form of a clear yellow solubilization zone formed around the colony, representing the production of organic acids as a possible mechanism of phosphate solubilization. This solubilization zone served as a qualitative indicator of the ability of the isolates to solubilize phosphate in the medium [23, 24].

For quantitative phosphate solubilization analysis, desired isolates were inoculated in triplicates into Pikovskaya's broth medium using 250 mL Erlenmeyer flasks. The flasks were then incubated for 7–14 days at 28°C on an incubator shaker at 180 RPM. Following the incubation period, the cultures were harvested by centrifugation at 6000 RPM for 15 min. A sterile, uninoculated medium served as the control in this study. The standard method described by Gaur [25] was employed to determine the remaining phosphate in the culture supernatant. This method likely involved colorimetric or spectrophotometric analysis to quantify the concentration of phosphate ions in the supernatant [25].

#### 2.3.2 Zinc Solubilization

All isolates underwent screening for zinc solubilization using a tris‐minimal salt medium mixed in deionized water. This medium was selected to assess the ability of rhizobacterial isolates to solubilize zinc oxide. Zinc oxide (ZnO) at a concentration of 0.2% was added to the medium as the sole source of zinc [26, 27]. The bacterial isolates were inoculated onto the designated medium and incubated at 28 ± 2°C for 7 days. Following incubation, the plates were examined for clear halo zones indicative of zinc solubilization. Each selected isolate's solubilization degree was evaluated by measuring the size of the solubilization zone around the colonies. Zinc solubilization in the broth medium was also studied further to assess the zinc solubilization capabilities of the isolates [28].

#### 2.3.3 Phytohormone Profiling of Potent PGPR Isolate—Conventional Method

Indole‐3‐acetic acid (IAA) was produced using a modified method based on Harikrishnan et al. [29] using the Salkowski reagent. Desired cultures were inoculated in L‐Tryptophan solution and incubated at 28°C in a rotary shaker at 130 RPM for 7 days. After the 7‐day incubation period, the solution was centrifuged at 11,000 RPM for 15 min. The supernatant obtained was then mixed with 2 mL of Salkowski reagent, which consisted of 1 mL of 0.5 M FeCl_3_ in 50 mL of 35% HClO_4_, and incubated for one h. The development of a pink coloration indicated the production of IAA. To quantify IAA, the absorbance of the solution was measured spectrophotometrically at 530 nm. A standard curve was constructed using known concentrations of IAA solutions to facilitate the quantification of IAA in the culture. An uninoculated medium with the reagent served as the control. The amount of IAA in the culture was expressed in micrograms per milliliter (µg mL^‐1^) [29].

Detection of gibberellins was carried out using TLC as a traditional approach. The isolates were cultivated in nutrient broth supplemented with tryptophan for 72 h at 28°C under shaking conditions. Following the incubation period, the supernatants were collected by centrifugation at 10,000 revolutions per minute (rpm) for 20 min [30]. Sample preparation involved acidification of the supernatant collected from the bacterial culture broth using 1 N HCl, resulting in a pH range of 2.5–3.0. Following acidification, the supernatant was subjected to extraction (twice a time) using a mixture of ethyl acetate and diethyl ether. The resulting extracts were then spotted onto a thin‐layer chromatography (TLC) sheet and allowed to run using a mobile phase consisting of butanol, acetic acid, and water in a ratio of 12:3:5 (v/v/v).

For the detection of cytokinin by traditional approach, TLC was used. The bacterial strains were cultured in M9 broth at 28°C in a rotary shaker at 150 RPM for 5 days. To obtain cell‐free supernatant, 50 mL of the culture was centrifuged at 16,000 RPM for 10 min at 4°C. The resulting supernatant was neutralized with 1 N NaOH to achieve a pH of 7.0, followed by lyophilization to dryness. The dried supernatant was then subjected to extraction (three times a time) using ethyl acetate. The organic phase obtained from each extraction was evaporated to dryness and reconstituted using methanol. Subsequently, the organic phase was dried and re‐dissolved in methanol. TLC was performed using a modified solvent system of n‐butanol, acetic acid, and water in a ratio of 12:3:5 (v/v/v) as the mobile phase. Following TLC, the Rf (retention factor) values were calculated for the spots developed on the TLC sheet [31].

#### 2.3.4 Phytohormone Profiling of Potent PGPR Isolate—Analytical Method

##### 2.3.4.1 Cytokinin Profiling by GC–MS (Gas Chromatography‐Mass Spectrometry)

Mass spectrometry is a technique used to separate ions or atoms of a compound based on their mass‐to‐charge ratio. Phytohormone production was detected on the 6th day of bacterial culture grown under shaking conditions. The process involved harvesting cell suspensions (50 mL) by centrifugation, followed by disruption of the cells using a centrifuge (Remi CM‐12 plus) and subsequent drying at 40°C. Perkin Elmer's GC‐Mass Spectrometer XL Turbomass system, including an autosampler and a quadrupole with a pre‐filter, was used to detect phytohormones. This mass spectrometer was equipped with Electron Ionization (EI) and Chemical Ionization (CI) sources, allowing for positive and negative ionization to obtain molecular weight information from complex samples. GC separation was performed using an HP‐5ms GC Column (30 m length, 0.25 mm ID, 0.25 µm film thickness, and 5‐inch format). The cytokinin was eluted isocratically with methanol/water containing 0.2% formic acid (50:50, v/v). The injector volume selected was 1 µL, and the injection temperature was maintained at 260°C. Helium was used as the carrier gas for GC, and the sample holding time was set to 15 min. The mass spectrometer data were analyzed usinga the National Institute of Standards and Technology (NIST) database, aiding in the identification and characterization of the phytohormones present in the samples [32].

##### 2.3.4.2 IAA Characterization by FTIR

Fourier transform infrared spectroscopy (FTIR) was employed as an analytical method to detect indole‐3‐acetic acid (IAA) from the 72‐h‐old culture supernatant. Three‐day‐old bacterial cultures served as the experimental group, while uninoculated media supplemented with L‐tryptophan served as the control. The separation process involved acidification of the samples to pH 2.5 using 1 N HCl, followed by extractions (three times a time) with ethyl acetate. The ethyl acetate fraction obtained was air‐dried before being re‐dissolved in methanol. Subsequently, 1 mg of the extract was diluted in 10 μL of methanol for FTIR spectroscopic analysis. For the FTIR analysis, the extract was applied as a thin film using a micro syringe onto clean flat discs with a diameter of 10 mm and a thickness of 2 mm and allowed to dry completely. Thermo Scientific Nicolet FTIR 6700 was used to record the FTIR spectral analysis of IAA in transmission mode within the 400–4000 cm^‐1^ range. The methanolic extract, which had been thoroughly dried, was mixed with spectral‐grade potassium bromide before the analysis [33, 34].

##### 2.3.4.3 Gibberellin Characterization by FTIR

Gibberellin production was proven using FTIR. The isolates were cultured in nutrient broth for 72 h at 28°C in a rotary shaker set to 90 rpm. After incubation, cell‐free supernatants were obtained by centrifugation at 10,000 rpm for 20 min. For gibberellin extraction, a separator funnel was employed. Specifically, 100 mL of supernatant was mixed with 250 mL of saturated NaHCO3 solution, and the organic phase was subsequently separated. The organic layer was acidified to pH 2.5 using 5 N HCl, and an equal volume of ethyl acetate was added to the funnel. The mixture was vigorously shaken for 5 min to facilitate gibberellin extraction. The ethyl acetate fraction was then separated and subjected to an additional extraction (2X) from the aqueous layer using 300 mL of ethyl acetate solvent each time. All ethyl acetate fractions were combined and dried over anhydrous Na_2_SO_4_. The resulting filtrate was dissolved in methanol in preparation for FTIR analysis. For FTIR spectroscopic analysis, a thin film was created using a micro syringe to inoculate the sample onto flat discs with a diameter of 10 mm and a thickness of 2 mm. Thermo Scientific Nicolet FTIR 6700 was utilized to record FTIR spectral analysis of gibberellins in transmission mode within the 400–4000 cm^‐1^ ranges in a methanolic extract combined with spectral‐grade potassium bromide [30, 33].

#### 2.3.5 HCN Production

All isolates underwent screening for hydrogen cyanide (HCN) production using the standard method developed by Lork [35]. Each isolate was streaked onto a nutrient agar medium supplemented with 4.4 gL^‐1^glycine. A Whatman number 1 filter paper was placed over the agar surface, pre‐soaked in a specific solution containing 0.5% picric acid and 2% sodium carbonate (w/v). The plates were then incubated at 28 ± 2°C for 4 days. During this incubation period, if the isolates were producing hydrogen cyanide (HCN), the presence of an orange or red color would develop on the filter paper. This color change serves as an indicator of HCN production by the bacterial isolates.

#### 2.3.6 Ammonia Production

To assess ammonia production, each rhizobacterial isolate was grown in peptone broth (10 mL) at 28 ± 2°C for 48 to 72 h. Following the incubation period, 0.2 mL of culture supernatantwas added with 1 mL of Nessler's reagen And the absorbance was measured spectrophotometrically at 450 nm. The concentration of ammonia was determined using an ammonium sulfate standard curve, which typically ranged from 0.1 to 1 µM mL^‐1^. By comparing the absorbance values of the samples to the standard curve, the ammonia concentration in the culture supernatants could be quantified accurately [36].

### 2.4 Polyphasic Characterization of Bacterial Isolate

#### 2.4.1 Morphological, Cultural, and Biochemical Characterization

According to Bergey's Manual for identification, the phenotypic and biochemical characteristics of isolate AB1 were assessed.

#### 2.4.2 Molecular Identification

Bacterial genomic DNA was isolated from the pure culture, and the quality of the DNA was assessed on a 1.0% agarose gel, revealing a single band of high‐molecular‐weight DNA. Subsequently, a fragment of the 16S rRNA gene was amplified using PCR. The PCR amplicon was then purified via column purification to eliminate contaminants. Following purification, DNA sequencing of the PCR amplicon was conducted using the BDT v3.1 Cycle Sequencing Kit with 357 F and 1391 R primers on an ABI 3500xl Genetic Analyzer. The resulting 16S rRNA sequence was utilized to perform a BLAST search against the National Center for Biotechnology Information (NCBI) Gene Bank database. The top 15 sequences were selected and aligned using multiple sequence alignment software based on the maximum identity score. The 16S rRNA sequence of strain AB1, spanning 716 base pairs (bp), was successfully sequenced and deposited in the GenBank database under the accession number MZ373174. Multiple alignments of the 716 bp 16S rRNA sequences and data from the NCBI library were used to construct a phylogenetic tree. The software program Mega version 10.2.6 was employed to calculate distances and perform clustering using the neighbor‐joining approach [37, 38, 39].

#### 2.4.3 Plant Growth Promotion Studies—Pot Assay

*Arachis hypogaea* L. (Groundnut) seeds of the GG20 variety obtained from Gujarat Bij Nigam Ahmedabad, India, were utilized as plant material in the study. The author owned the seeds, and all experiments were conducted following the relevant guidelines and regulations of the institute. Two distinct treatments were employed for analysis: T1 involved the seed priming method, while T2 utilized direct inoculation via liquid bio‐fertilizer spray. Both treatments were applied to nine isolates, and two individual controls were included. Evaluations were performed with six replicates. Healthy seeds were surface sterilized with 0.1% HgCl_2_ solution for 2 min, followed by rinsing six times with sterile distilled water. Subsequently, different isolates were cultured individually in nutrient broth on an incubator shaker (180 rpm) at 28 ± 2°C for 24 h. Following incubation, the seeds were coated with 1% carboxymethylcellulose (CMC) as an adhesive for subsequent treatment application [40].

Following treatment with the isolates or seed priming for 30 min, the groundnut seeds were sown into sterilized pots. Five seeds were sown in each pot containing soil. The pots were positioned to receive adequate sunlight and maintain suitable temperatures conducive to seed germination. Subsequently, irrigation was performed daily using sterilized water to ensure optimal moisture levels for germination. A seed germination study was conducted by monitoring and recording germination rates daily. This involved calculating the percentage of seeds that had germinated each day, allowing for the assessment of the effectiveness of the treatments in promoting seed germination and early seedling growth [41].

After 21 days of sowing, the plants were carefully uprooted and washed by dipping them into a vessel to remove any soil or debris adhering to the roots and foliage. Subsequently, plant height (in cm) and root length (in cm) were recorded for each plant. The shoot and root's dry weights were determined after drying the plant material in an oven at 70°C for 1 day. This process ensured accurate measurements of plant biomass. The experimental procedure was repeated twice to validate the results and ensure consistency. Additionally, observations were recorded on various parameters, including the seedling emergence rate, chlorophyll content, leaf area, and overall plant vigor. Random samples were drawn from the experimental population to capture variability and ensure representative data collection across the plant population [42].

## 3 Results

### 3.1 Isolation of Rhizospheric Bacteria

In this study, 11 distinct isolates were successfully isolated and designated as follows: AB1, AB3, ACP, CAB3, AB5, AB6, AB7, CAB1, AB8, AB9, and AB10. These isolates were then evaluated as plant growth‐promoting rhizobacteria (PGPR) using various traits associated with PGPR activity, as outlined in the study.

### 3.2 Plant Growth‐Promoting Traits

#### 3.2.1 Phosphate Solubilization

**Figure 2:**
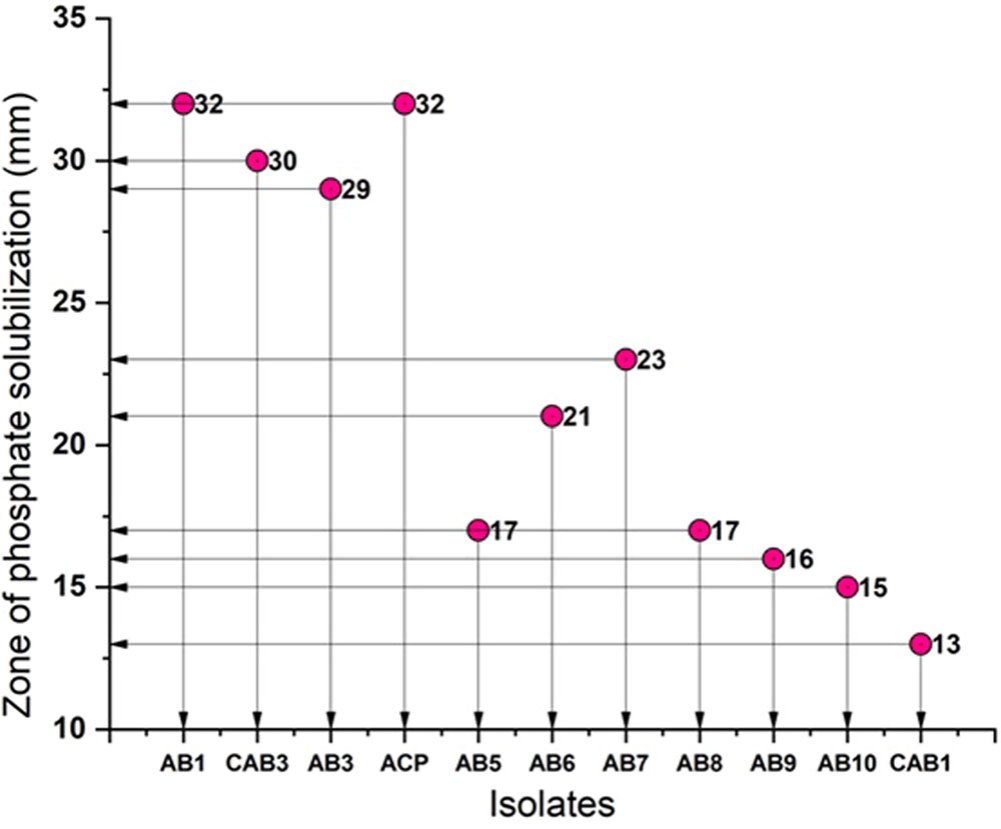
Zone of phosphate solubilization (in mm) for rhizospheric isolates.

The zone of phosphate solubilization on solid Pikovskaya's mediumranged between 15 and 31 mm for all isolates, as depicted in (Figure 2). Notably, isolated AB1 exhibited a maximum zone of 31 mm within 4 days of incubation.

**Figure 3:**
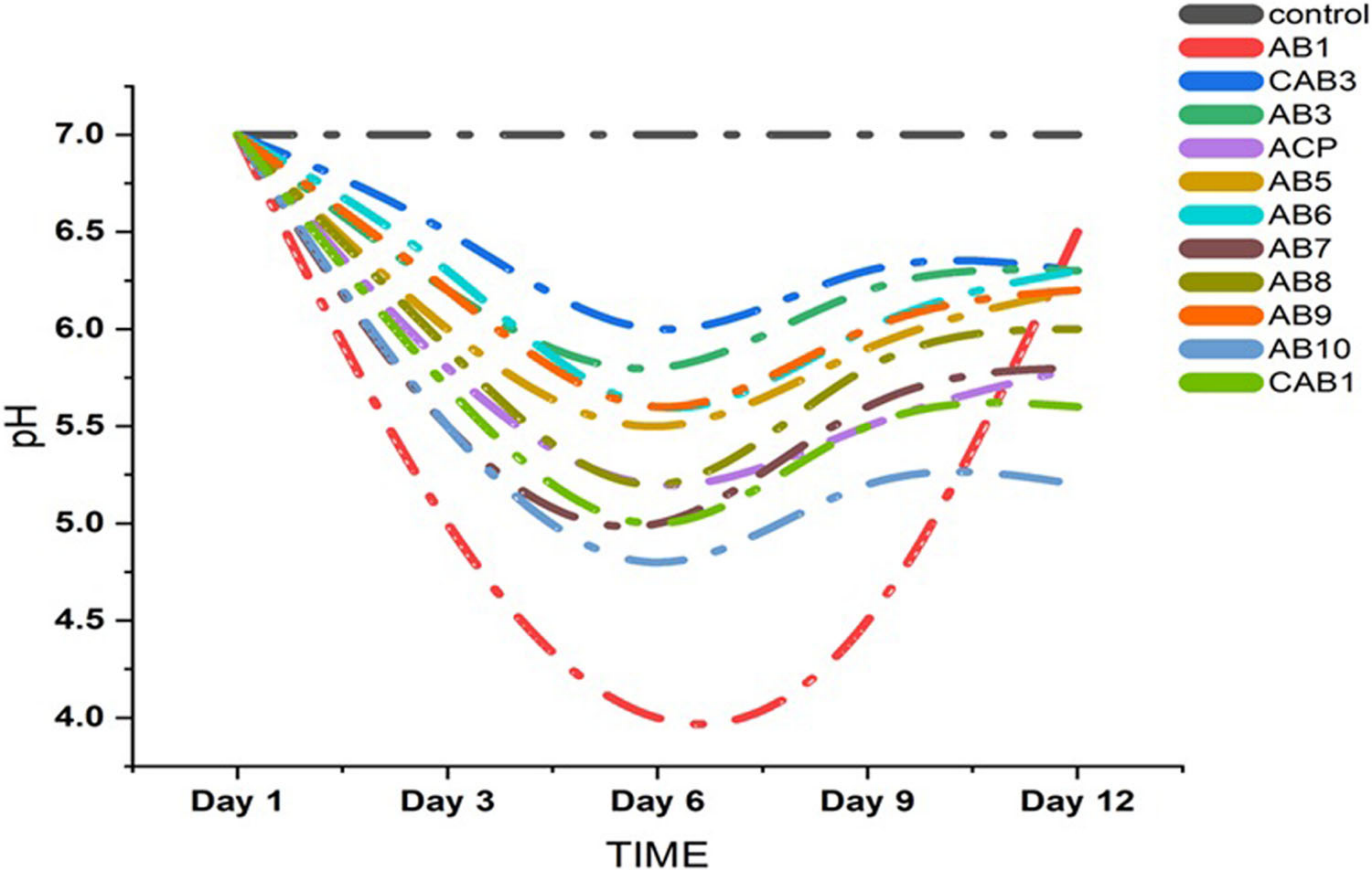
Qualitative analysis of Phosphate solubilization by estimating pH at various time intervals.

In the liquid PVK medium, a decrease in pH from neutral to acidic was observed (Figure 3). The isolated strain AB1 also demonstrated the maximum phosphate solubilization in the medium within a week. In the context of phosphate solubilization in PVK broth medium, isolate AB1 exhibited a remarkable 56.44 g mL^‐1^ of phosphate solubilization on day 12.

#### 3.2.2 HCN Production

**Figure 4:**
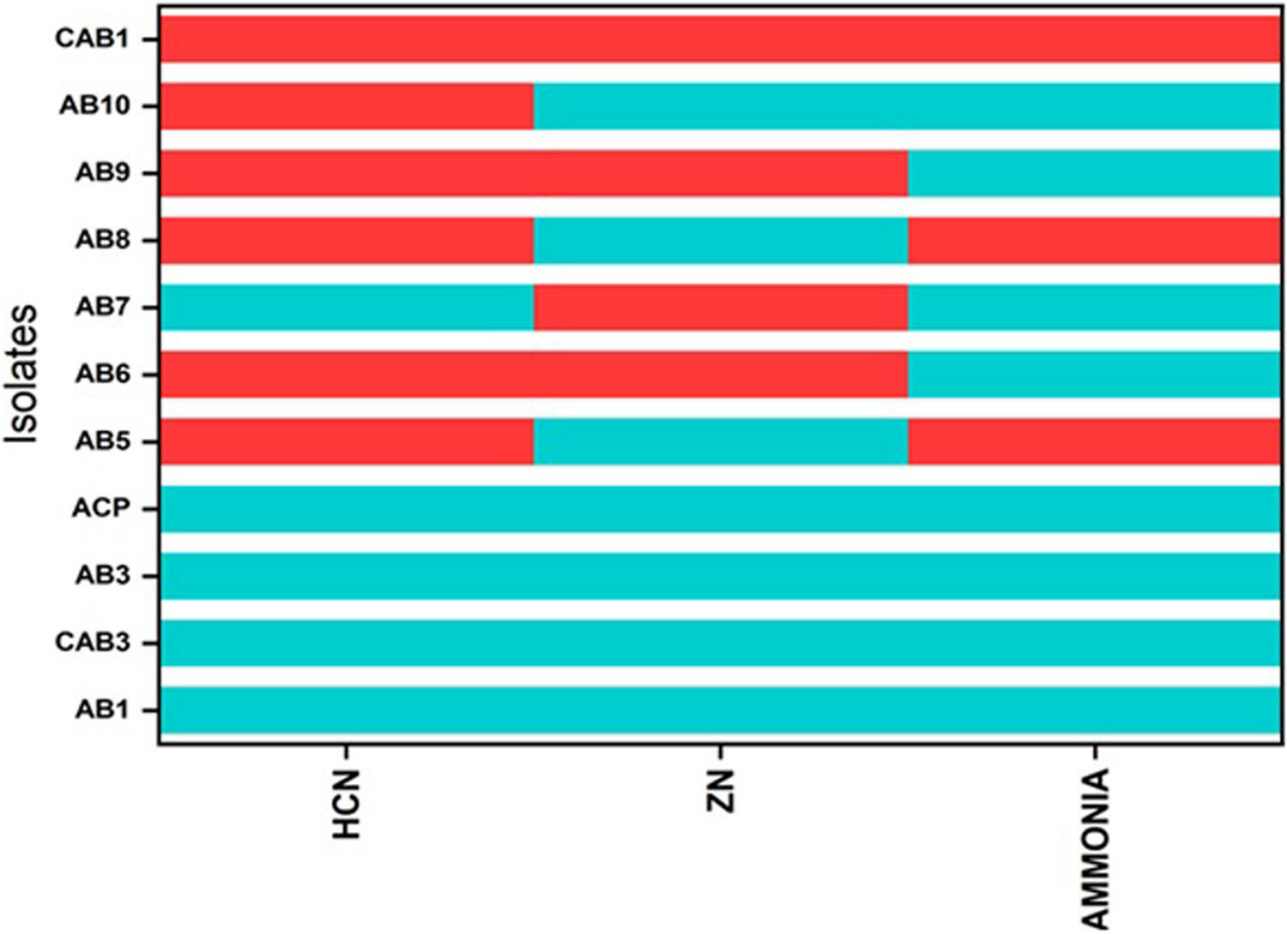
Qualitative results of the production of HCN and ammonia and Zinc solubilization. The red color shows negative while the blue color shows positive results.

Five isolates demonstrated the ability to produce hydrogen cyanide (Figure 4), as indicated by a discernible change in the color of the filter paper from yellow to brown. Specifically, isolates AB1, CAB3, AB3, ACP, and AB7 exhibited positive results for HCN production, while AB5, AB6, AB8, AB9, AB10, and CAB10 yielded negative results.

#### 3.2.3 Zinc Solubilization

**Figure 5:**
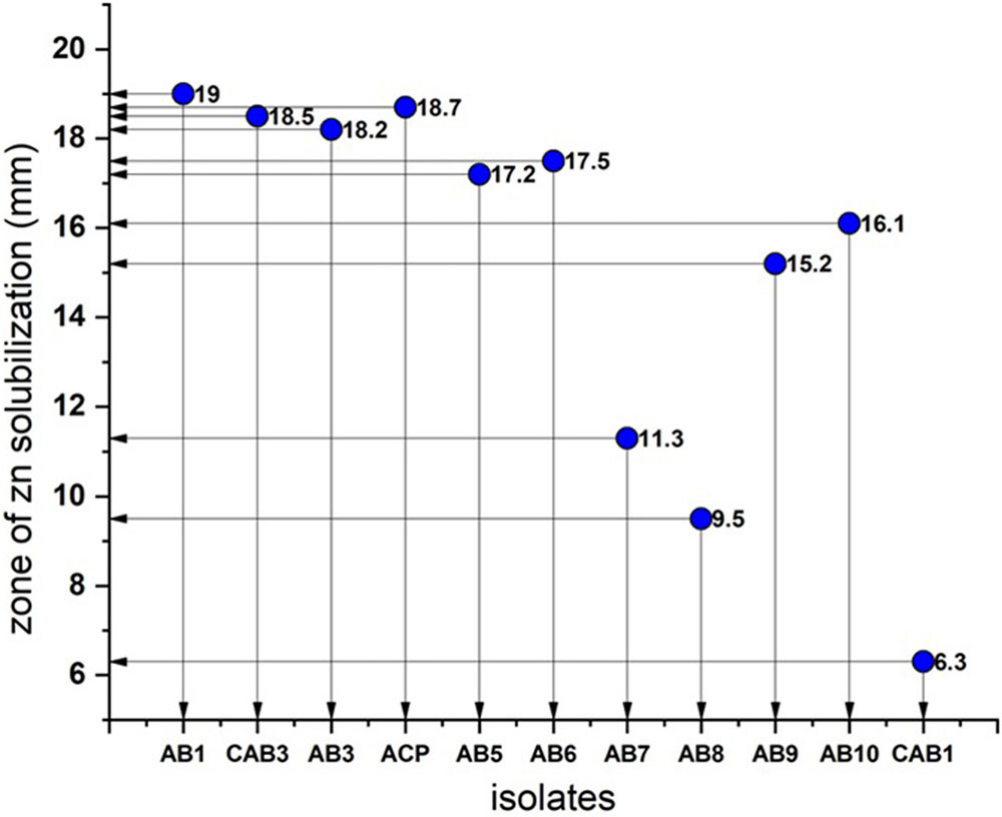
Zone of zinc solubilization (in mm) for rhizospheric isolates.

**Figure 6:**
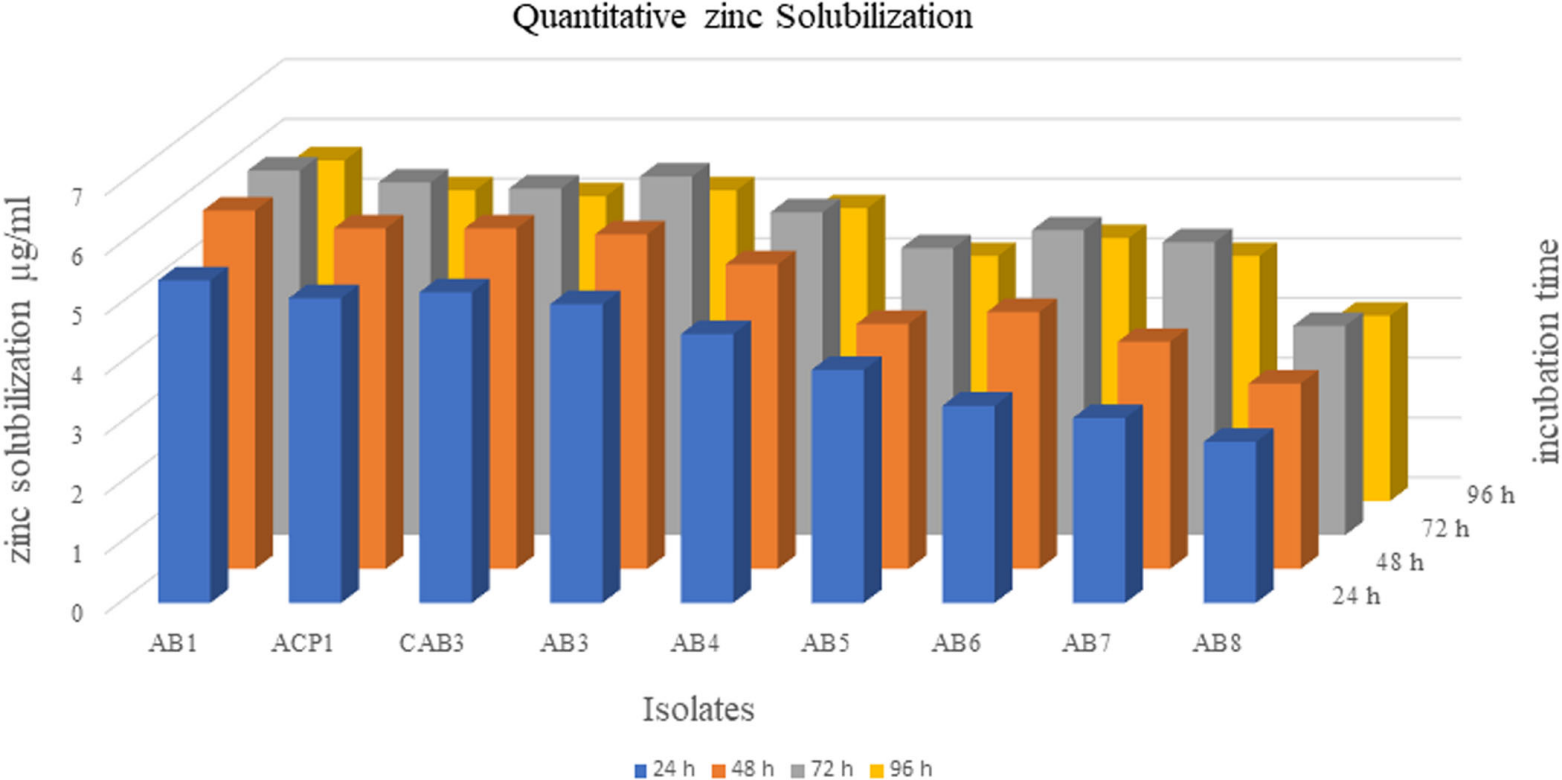
Quantitative zinc solubilization.

Among the isolates studied, all except AB6, AB7, and AB9 exhibited positive results for zinc solubilization. Notably, isolate AB1 demonstrated a maximal Zone of Solubilization (ZOS) of 19 mm. In addition, it displayed 6.1 µg mL^−1^ of zinc solubilization at 72 h in the broth medium, as determined by quantitative assay (Figures 5 and 6).

#### 3.2.4 Ammonia Production

**Figure 7:**
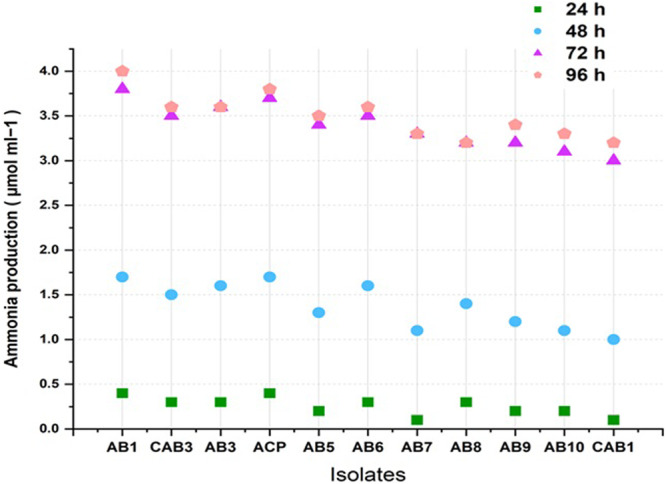
Ammonia Production (µ mL^‐1^) by rhizospheric isolates.

In the context of ammonia production, all isolates except AB5, AB8, and CAB1 yielded positive results. AB1 exhibited the highest ammonia production in quantitative analysis conducted at 24, 48, 72, and 96 h. ACP, AB3, CAB3, AB6, and AB5 also demonstrated notable results for ammonia production, as illustrated in (Figure 7).

### 3.3 Bacterial Phytohormones Characterization Using Traditional Methods

**Figure 8:**
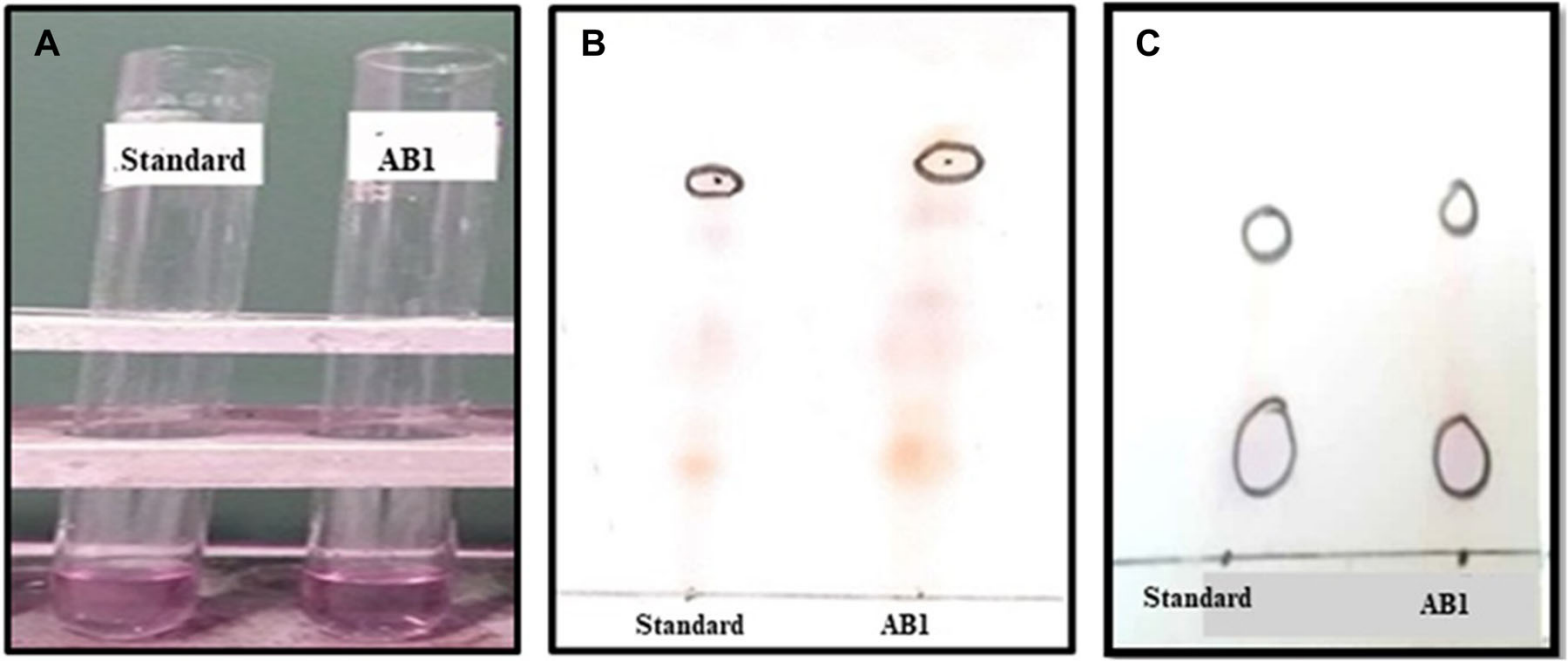
Phytohormone production (IAA, Gibberellins, and Cytokinins) by a traditional method where (A) The production of the pink color of standard IAA. (B) TLC run of gibberellins for AB1 as almost similar to standard, and (C) TLC run of cytokinins for AB1 and standard.

The rhizobacterial plant growth‐promoting trait of phytohormone production was investigated for three main phytohormones: Indole acetic acid (IAA), Gibberellins, and cytokinins. IAA production, a key property of PGPR that stimulates plant growth, was assessed using the Salkowski reagent, which resulted in a visible color change (Figure 8). IAA production was quantitively measured at 530 nm. In our study, TLC analysis of a purified and extracted sample from isolated strain AB1 revealed a spot with an RF value of 0.61 (Figure 8).

Screening for cytokinin production by rhizobacteria indicated that isolate AB1 exhibited an RF value of 0.60 (Figure 8) after running TLC of the extracted sample from the M9 medium.

### 3.4 Bacterial Phytohormones Characterization Using Analytical Methods

#### 3.4.1 IAA Characterization

**Figure 9:**
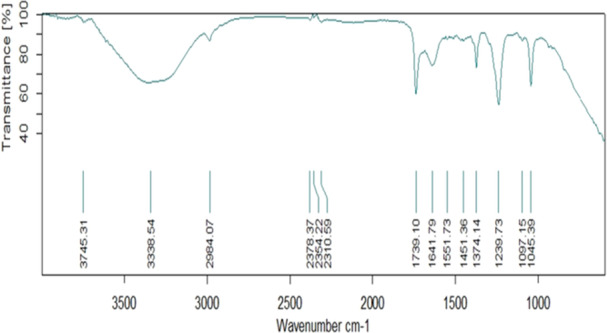
FTIR spectra for IAA production by *Bacillus rugosus* AB1.

The FTIR spectra of extracted Indole Acetic Acid (IAA) from AB1 revealed characteristic peaks corresponding to specific molecular bonds. The (N–H) stretching of the indole fraction was observed at 3338 cm^−1^, while the (N–H) bending occurred at 1641 cm^−1^ for the (C–N) bond of the indole ring. In the aromatic ring, (C–H) stretching and bending were observed at 2984 cm^−1^. Additionally, (C = C) stretching and C‐H bending were observed at 1551 cm^−1^ and 1451 cm^−1^, respectively. The strong bond of (C = O) in IAA was identified at 1739 cm^−1^ (Figure 9).

### 3.5 Gibberellin Characterization

**Figure 10:**
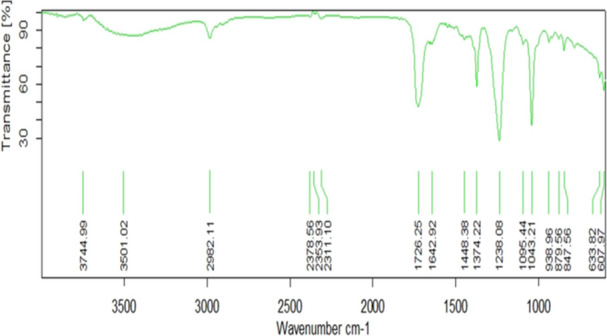
FTIR spectra for GA production by *Bacillus rugosus* AB1.

The FTIR spectrum of AB1 demonstrates a peak at 3744.99 cm^‐1^ and 3501.02 cm^−1^ for (O–H) medium and strong stretching, respectively (Figure 10), indicating the vibration of the hydroxyl group of a carboxylic acid (COOH). This characteristic hydroxyl group is attached to carbon 7 of the gibberellin structure. A peak at 2982.11 cm^−1^ and 1448.38 cm^−1^ for the aromatic ring represents the (‐C‐H) medium stretching and bending, respectively.

The gibberellin spectrum indicates a methyl (‐CH) group attached to carbon 4 of the ring. A distinct peak at 1642.92 cm^‐1^ with stretching and 879.56 cm^−1^ with bending in the spectrum is characteristic of the alkenes group (C = C). The peak at 1726.25 cm^−1^ also represents the (C = O) with strong stretching. Stretching for the (C–O) bond was observed at 1238.08 cm^−1^ and 1095.44 cm^−1^, and a strong stretching for (C–C) was observed at 8456 cm^−1^.

#### 3.5.1 Cytokinins Characterization

**Figure 11:**
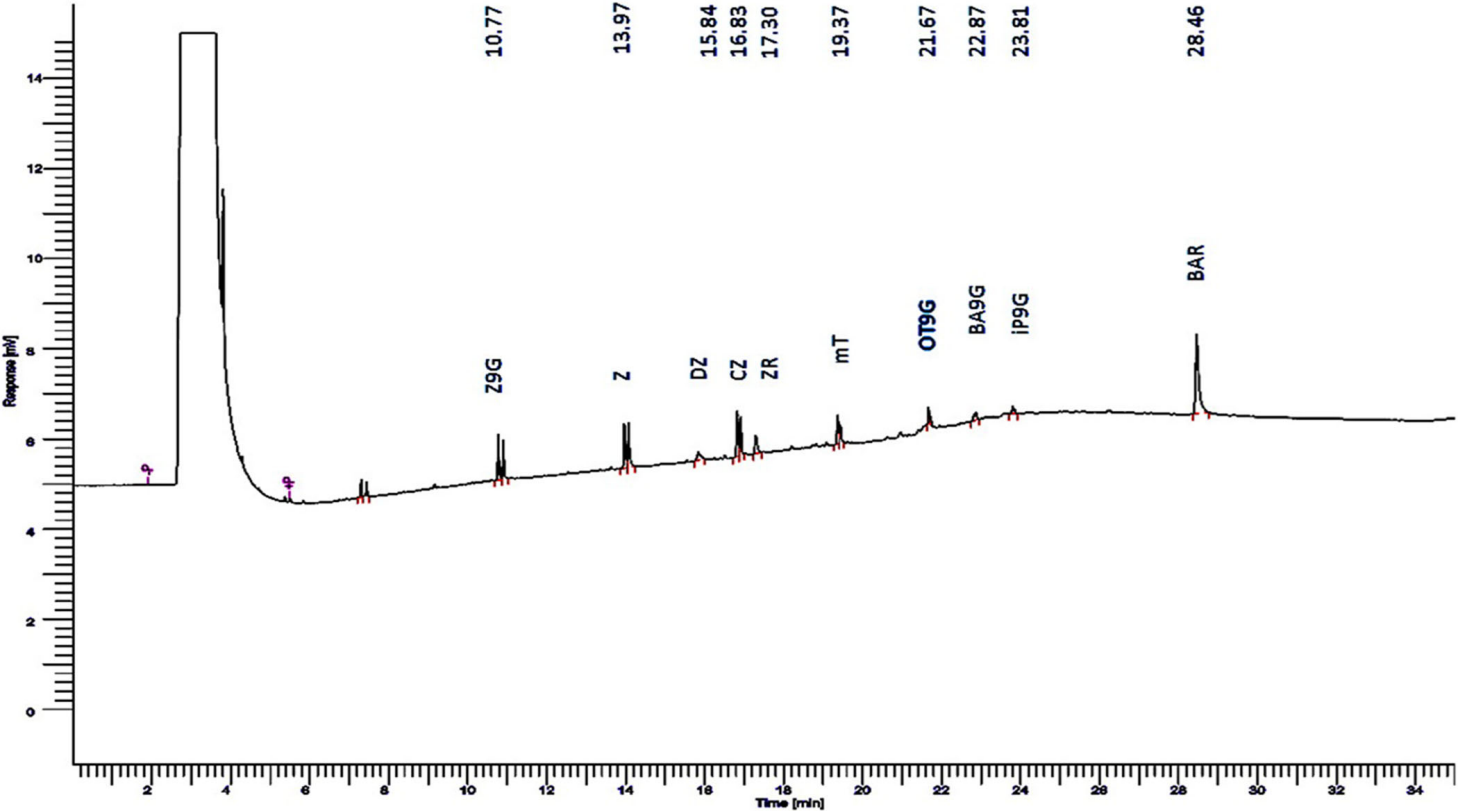
Peaks showing presence of cytokinin components in chromatogram of GC‐MS (Z9G = transzeatin 9 glucoside, BA9G = benzylaminopurine 9 glycoside, BAR = benzylaminopurine riboside, CZ = cis zeatin, DZ = dihydro zeatin, iP9G = isopentyladenine 9‐ glucoside, mT = meta topolin, OT9g = orthotopolin 9 glucoside, Z = trans zeatin, ZR = trans zeatin riboside.

Gas chromatography with mass spectrometry (GC‐MS) has been utilized for cytokinin analyses since the 1990s. Cytokinins, being non‐volatile compounds, require derivatization to enhance their volatility for GC‐MS analysis. The structures of nearly all naturally occurring cytokinins have been elucidated through GC‐MS analysis, as reported by Dobrev and Kamınek (2002). Chromatogram of GC‐MS analysis (Figure 11) demonstrates the cytokine components in the sample of AB1 include Z9G, Z, DZ, CZ, ZR, mT, OT9G, BA9G, Ip9G, and BAR, with respective RT values of 10.77, 13.97, 15.84, 16.83, 17.30, 19.37, 21.67, 22.87, and 23.81.

### 3.6 Bacterial Identification

#### 3.6.1 Morphological, Cultural, and Bio‐Chemical Identification

Among the 11 rhizospheric isolates, AB1 AB1 was identified as the most efficient isolate. The cultural, microscopic, and biochemical characteristics of isolate AB1 closely resembled those of *Bacillus* sp. (Supporting Information S1: Table 1).

#### 3.6.2 Molecular Identification

**Figure 12:**
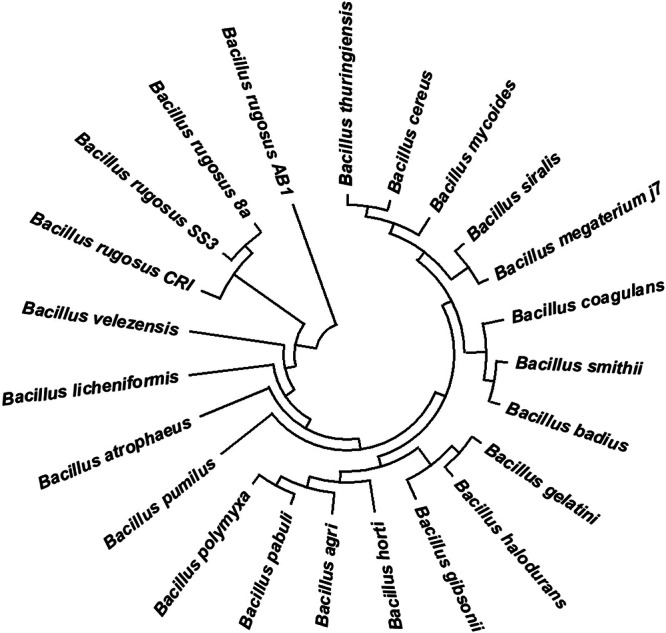
Phylogenetic tree created by MEGA 10.2.6 software to present the relationship between closely related species of *Bacillus rugosus AB1*.

The phylogenetic tree, constructed using MEGA software v‐10.2, illustrates branching points with bootstrap values based on 1000 replications reported as percentages. In the neighbor‐joining analysis, strain AB1 forms a distinct branch and shares a 99% sequence similarity with *Bacillus tequilensis MA‐67*, *Bacillus subtilis subsp. stercoris CHD*, *Bacillus amyloliquefaciens R4.6*, and *Bacillus methylotrophicus OrH30* (Figure 12).

Thus, isolate AB1 was identified as *Bacillus rugosus AB1*, and its 16S rRNA gene sequences were deposited to NCBI Gene Bank with Accession No. MZ373174.

### 3.7 Plant Growth Promotion Study—Pot Assay

**Figure 13:**
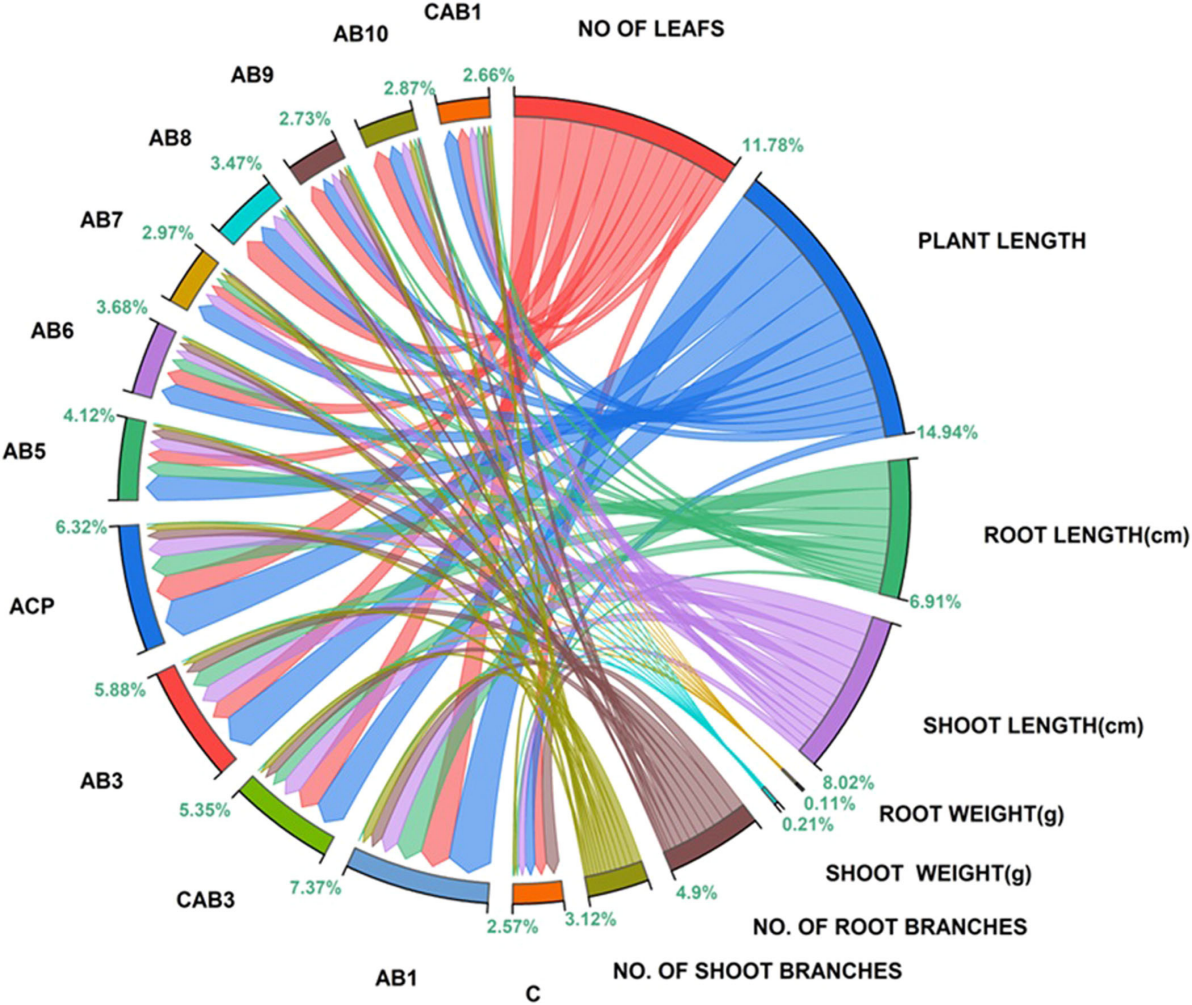
Categorical graph representing pot analysis with study parameters and their interaction with individual isolates in the form of Nodes.

**Figure 14:**
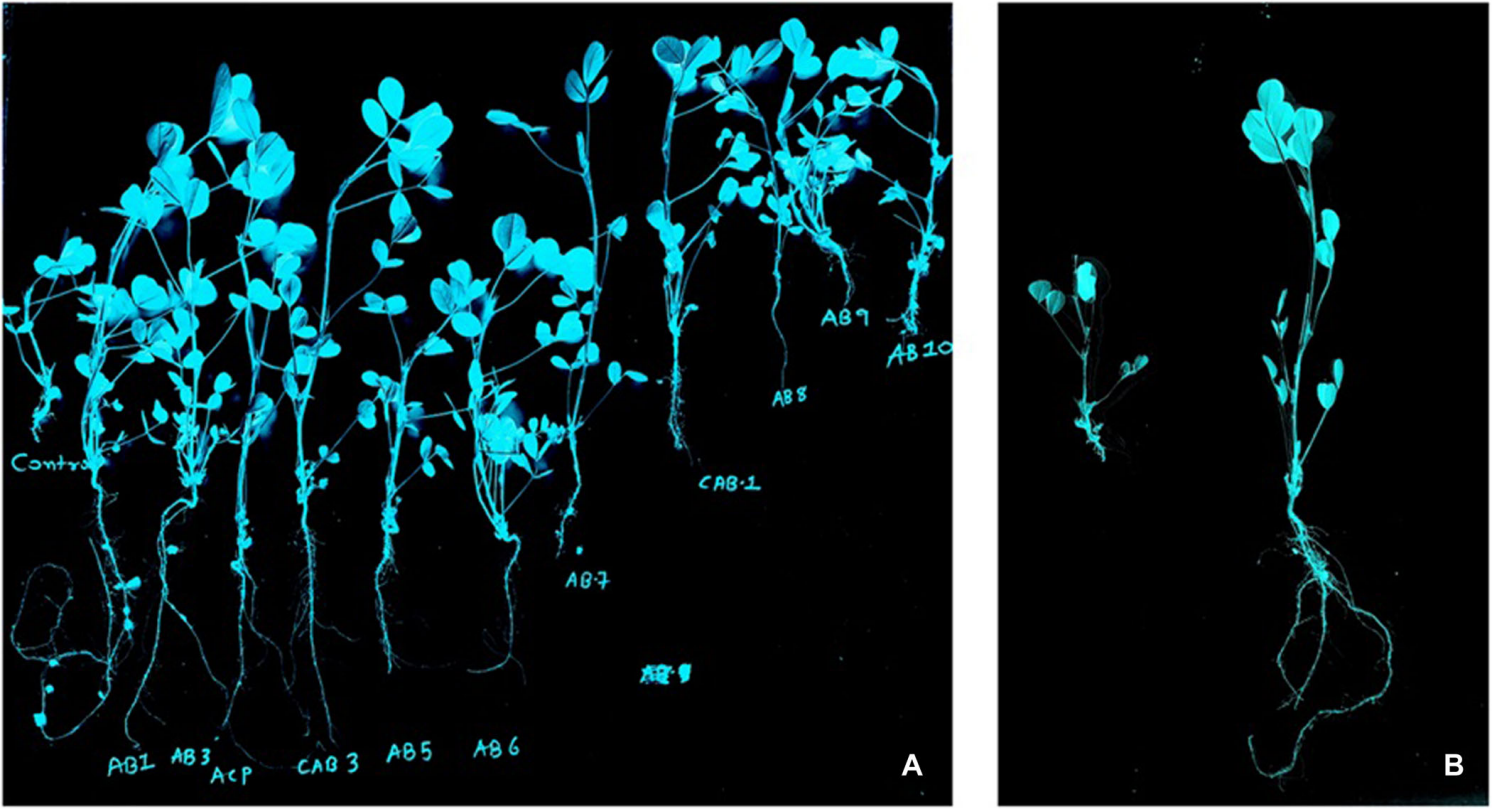
Radiographic images of pot study of *Arachis hypogaea L*. Image (A) showed comparative results of all isolates against control, and image (B) shows *Bacillus rugosus* AB1 against control.

The pot experiment results indicate that seeds bacterized with strain AB1 enhanced plant growth parameters in *Arachis hypogaea L*. compared to the other eight isolates (Figures 13, 14). Data are categorized into two main categories: different isolates and various pot study parameters. Each parameter is represented by a node color directly correlating with the isolates category. Larger node sizes indicate higher results associated with isolates, with AB1 showing the maximum categorical result of 7.37% among other isolates and the yield of *Arachis hypogaea L* calculated for both T1 and T2 treatments.

**Figure 15:**
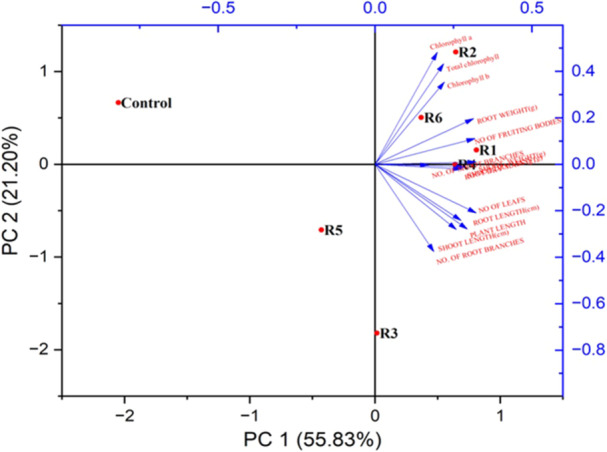
Principal component analysis of six replicates of *Bacillus rugosus* AB1 in T1 treatment of pot trials.

The growth enhancement of *Arachis hypogaea L*. by PCA compared to control plants and other isolates indicated the biopotential of *Bacillus. rugosus* (Table 1 and Figure 15). Each value represents the mean of six replicas. Plot PC1 shows a value of 55.83%, and PC2 shows 21.20% similarities. Root length, shoot length, root branch, shoot branch, root weight, shoot weight, and chlorophyll contents are more definite compared to other isolates in AB1.

**Table 1:** Yield of *Arachis hypogaea L* in T1 and T2 treatments.

AB1	Pods/plant	Kernels/pod	Pod yield (kg/ha)	Kernel yield (kg/ha)	Total yield (kg/ha)	Yield compared to control (%)
C	15	1.85	2165	1472	3637	
T1 (Seed priming)	32	2.25	3388	2286	5674	43.75%
T2 (Liquid biofertilizer spray)	28	2.12	3186	2164	5350	38.12%

## 4 Discussion

Rhizosphere harbors a wide range of rhizobacteria, mostly PGPR, that produce a wide range PGP traits to support plant growth. *Bacillus* spp. is among the major groups of soil PGPR present in various rhizospheres. Alkilayh et al. [43] have isolated *B. subtilis* from tomato rhizosphere. Sagar et al. [42] have isolated two potent bacilli PGPR strains, namely *B. subtilis* PR30 and PR31, from tomato, broccoli, and chickpea rhizosphere. Manasa et al. [44] isolated Bacillus sp. from sorghum rhizosphere. Eswaran et al. isolated *Bacillus amyloliquefaciens* BA01 from groundnut rhizosphere.

Phosphate‐solubilizing bacteria (PSB) employ the production of organic acids as one of the fundamental mechanisms for phosphate solubilization [13, 17, 26]. Phosphate solubilization by bacterial isolates is also attributed to gluconic acid, 2 keto‐gluconic acid and phosphatase production [45, 46]. Results of the present study indicated that all the bacterial isolates solubilize phosphates.

A decrease in the pH of PKV media from neutral to acidic indicated organic acid production. This shift in pH suggests a correlation between the production of organic acid and the solubilization of phosphate in the medium. A study on tri‐calcium phosphate solubilization in a liquid medium by *Bacillus* sp. and *Pseudomonas* sp. (P1) demonstrated similar trends. The maximum phosphate solubilization by strain AB1 highlights its potential mechanism for promoting rapid plant growth. Maximum phosphate solubilization in PVK broth by isolate AB1 aligns with the study by Jha et al. [24], who reported 22 g mL^−1^ of P solubilization by *Enterobacter cancerogenus* MSA2 in a liquid medium.

The formation of hydrogen cyanide represents a significant mechanism for stimulating plant growth [47]. A discernible change in the color of the filter paper from yellow to brown indicates hydrogen cyanide (HCN) by the isolate. Similar observations regarding HCN production by *Pseudomonas* sp. isolates on King's B agar were reported by Sharma et al. [48], where the color changed from deep yellow to orange‐brown after 4 days at 28°C. These findings underscore the variability in HCN production among different bacterial isolates and highlight the importance of this mechanism in promoting plant growth.

Zinc is a critical element for plant growth and is essential for various physiological processes. Zinc deficiency represents one of the most prevalent micronutrient deficiencies encountered in crops worldwide, leading to substantial yield losses [49]. Zinc‐solubilizing bacterial strains have been shown to enhance root length, root hair, and surface area, thereby improving nutrient availability, as Hussain et al. reported [50].

Ammonia is a vital nitrogen source for plants, crucial for promoting plant growth and enhancing fruit and seed production, thus leading to increased yields [51]. According to Goswami et al. [33], a time‐dependent investigation of ammonia production in *Kocuria turfanensis* 2M4 showed that it produced 1.8 µM mL^−1^ of NH_3_ after 72 h of incubation at 30 ± 2°C under shaking conditions. Production of 3.8 µM mL^−1^ at 72 h of incubation at 28°C by isolate AB1 is significantly higher levels of ammonia production than that reported by Goswami et al. [33].

The rhizobacterial plant growth‐promoting trait of phytohormone production was investigated for three main phytohormones: Indole acetic acid (IAA), Gibberellins, and cytokinins. IAA production is a key property of PGPR that stimulates plant growth. All the isolates produced reasonable amounts of IAA. Gibberellins (GAs) represent a class of carboxylic phytohormones that govern growth and influence various developmental processes in plants. Macmillan et al. have effectively classified nine types of GAs based on thin‐layer chromatography (TLC) analysis. TLC analysis of a purified and extracted sample from isolated strain AB1 revealed resembled the RF value of GAs type 5, as reported by MacMillan & Suter [52].

Cytokinin production by rhizobacteria significantly enhances plant growth. (Hussain & Hasnain [31] reported cytokinin production from *Bacillus licheniformis Am2*, *Bacillus subtilis BC1*, and *Pseudomonas aeruginosa E2* with an RF value of 0.58. RF values of the extract from cytokine production by strain AB1 are comparable to the findings reported by Hussain & Hasnain, suggesting similar cytokinin production characteristics [31].

The FTIR spectra of extracted Indole Acetic Acid (IAA) from isolate AB1 exhibited characteristic peaks corresponding to specific molecular bonds. The observed peaks of the extracted IAA align with the distinctive peaks of IAA, indicating IAA's presence in the medium.

The FTIR spectrum showing peaks at 3744.99 cm^−1^ and 3501.02 cm^−1^ for (O‐H) medium and strong stretching, respectively, indicated the vibration of the hydroxyl group of a carboxylic acid (COOH). This characteristic hydroxyl group is attached to carbon 7 of the gibberellin structure. Comparison of FTIR data of isolate AB1 with the spectrum available in the spectra databases (SDBS) confirmed the presence of IAA.

Gas chromatography with mass spectrometry (GC‐MS) has been utilized for cytokinin analyses since the 1990s. Cytokinins, being non‐volatile compounds, require derivatization to enhance their volatility for GC‐MS analysis. The structures of nearly all naturally occurring cytokinins have been elucidated through GC‐MS analysis, as reported by Dobrev and Kamınek [53]. The components detected in the sample of isolate AB1 with respective RT values indicate essential cytokinin constituents, as reported by Tarkowski et al. [54].

The oThe first report is the occurrence of Bacillus rugosus AB1 as multifarious PGPR in groundnut rhizosphere. *Bacillus* spp. is one of the major PGPR reported to promote the growth of a wide range of plants. Sagar et al. [13] isolated *B. subtilis* from organic farms and demonstrated itsgrowth‐promoting potential in tomato, broccoli, and chickpea. Eswaran et al [2] showed the plant growth‐promoting potential of *Bacillus amyloliquefaciens* BA01 in groundnut. Manasa et al. [44] claimed co‐inoculation of *Bacillus* spp. for growth promotion and iron fortification in sorghum. Nithipriya et al. [3] found that siderophore and other PGP‐producing *Bacillus subtilis* improve sesame plant growth and oil content. Kishore et al. [55] reported plant growth promotion in *A. hypogea* (L.) following the inoculation of Bacillus species isolated from the phylosphere. Mohite et al. [56] isolated and characterized IAA‐producing *Bacillus* sp. from isolated IAA‐producing strains of *Bacillus* sp., namely, *B. megaterium, Lactobacillus casei, B. subtilis, B. cereus* and *Lactobacillus acidophilus* from rhizosphere soil and found these isolate to promote wheat growth.

Countless studies have focused on strains belonging to *Bacillus sp*., highlighting their significant role as effective bio‐fertilizers due to their diverse plant growth‐promoting activity [44, 45, 46, 57]. However, the plant growth‐promoting (PGP) activity of the isolated strain of *Bacillus rugosus* AB1 has not been extensively explored. In the present study, we isolated *Bacillus rugosus* AB1 from the rhizospheric soil of the central Gujarat region, revealing diverse PGP potentials such as phosphate solubilization, zinc solubilization, and production of ammonia, HCN, and phytohormone through both traditional and analytical methods. The screening and characterizing this rhizobacteria from the rhizosphere of healthy *Arachis hypogaea L*. plants represent an ecological opportunity to enhance soil fertility and biomass production on peripheral lands. This study suggests that the rhizobacterial strain *Bacillus rugosus* AB1 is a suitable bio‐fertilizer. It warrants exploration for its bio‐control potential to promote plant growth under various stress conditions in groundnut cultivation. Such indigenous PGPR could offer an environment‐friendly solution for soil health improvement and increased crop production.

## Author Contributions

**Aniruddh Rabari** and **Janki Ruparelia:** methodology, investigation, and writing ‐ original draft. **Chaitanya Kumar Jha:** conceptualization, supervision, and project administration. **Kahkashan Perveen, Abhijit Debnath, Maheswari Behera** and **Andrea Mastinu:** writing – review and editing and formal analysis.

## Ethics Statement

The authors have nothing to report.

## Consent

The authors have nothing to report.

## Conflicts of Interest

The authors declare no conflicts of interest.

## Supporting information

756SupportingInformation.

## Data Availability

All the study data are included in the manuscript and supplementary files.
