# Supplementary material for: Evaluating the Phytohormone Proficiencies of Multifarious Bacillus rugosus for Growth Promotion in Arachis hypogaea (L.)
Source: J Basic Microbiol. 2025 Apr 3;65(6):e70011. doi: 10.1002/jobm.70011 (PMC12134843; doi:10.1002/jobm.70011)
Supplement: Supplementary file 1 — 756SupportingInformation. [file JOBM-65-e70011-s001.docx]

| **Characteristics** | ***Bacillus rugosus* *AB1*** |
| --- | --- |
| Urea Test, Citrate Utilization, Phenylalanine Test, Triple Sugar Iron test (Gas), Lipid hydrolysis test, pigmentation, | (-) |
| Methyl Red, Voges-Proskauer, Ammonia production, Triple Sugar Iron test (acid), Starch hydrolysis test, Casein hydrolysis test, Indole production test, Motility, Catalase test, Dehydrogenase test, oxidase, Gelatin test | (+) |
| Sugar utilization  Glucose , Fructose, Sucrose, Lactose, Mannitol, Xylose, D-mannose, L-Arabinose, | (+) |

**Table S1** Biochemical characteristics of isolates
